# 2023 European Society of Cardiology guidelines on the management of cardiomyopathies: Statement of endorsement by the NVVC

**DOI:** 10.1007/s12471-025-01955-2

**Published:** 2025-04-14

**Authors:** Judith A. Groeneweg, Bas M. van Dalen, Moniek P. G. J. Cox, Stephane Heymans, Richard L. Braam, Michelle Michels, Folkert W. Asselbergs

**Affiliations:** 1 Department of Cardiology, Diakonessenhuis, Utrecht, The Netherlands; 2 Department of Cardiology, Franciscus Gasthuis and Vlietland, Rotterdam, The Netherlands; 3 Department of Cardiology, Cardiovascular Institute, Thoraxcentre, Erasmus MC, Rotterdam, The Netherlands; 4 Department of Cardiology, University of Groningen, University Medical Centre Groningen, Groningen, The Netherlands; 5 Department of Cardiology, Cardiovascular Research Institute Maastricht, University of Maastricht & Maastricht University Medical Centre, Maastricht, The Netherlands; 6 Department of Cardiovascular Sciences, Centre for Vascular and Molecular Biology, KU Leuven, Leuven, Belgium; 7 Department of Cardiology, Gelre Hospitals, Apeldoorn, The Netherlands; 8 Department of Cardiology, Amsterdam University Medical Centres, University of Amsterdam, Amsterdam, The Netherlands; 9 Health Data Research UK and Institute of Health Informatics, University College London, London, UK

**Keywords:** Cardiomyopathy, Cardiogenetics, Risk stratification, Management

## Abstract

This article contextualises the 2023 European Society of Cardiology (ESC) guidelines for the management of cardiomyopathies for clinical practice in the Netherlands. The guideline addendum provides additional recommendations for situations where the ESC guidelines may not fully align with Dutch clinical practice. By endorsing the ESC guidelines through this addendum, the Netherlands Society of Cardiology (*Nederlandse Vereniging Voor Cardiologie*) supports its members in adhering to evidence-based management strategies for cardiomyopathies. As Dutch cardiologists generally adopt the ESC guidelines quickly, this contextualisation is essential for effective application thereof within the Dutch healthcare setting.

## Introduction

Cardiogenetics is a dynamic and rapidly evolving domain within the field of cardiology. With increasing knowledge on phenotypes, genetic background and specific treatment options for each specific type of cardiomyopathy, it is of utmost relevance for every cardiologist to familiarise themself with the principles of cardiomyopathy care. Ensuring access to multidisciplinary cardiogenetics expertise—whether through dedicated centres or shared-care models—is essential for optimising the management of cardiomyopathy patients. The European Society of Cardiology (ESC) guidelines offer a wealth of background information, specific management and follow-up recommendations for clinical practice and, very importantly, guidance on how to provide multidisciplinary cardiogenetics care at the individual and organisational level [1].

## Summary and translation into clinical practice in the Netherlands

### Phenotypic approach to cardiomyopathies

Cardiomyopathy is defined as a myocardial disorder in which the heart muscle is structurally and functionally abnormal, in the absence of significant coronary artery disease, hypertension, valvular disease and congenital heart disease sufficient to cause the observed myocardial abnormality [1].

**Fig. 1:**
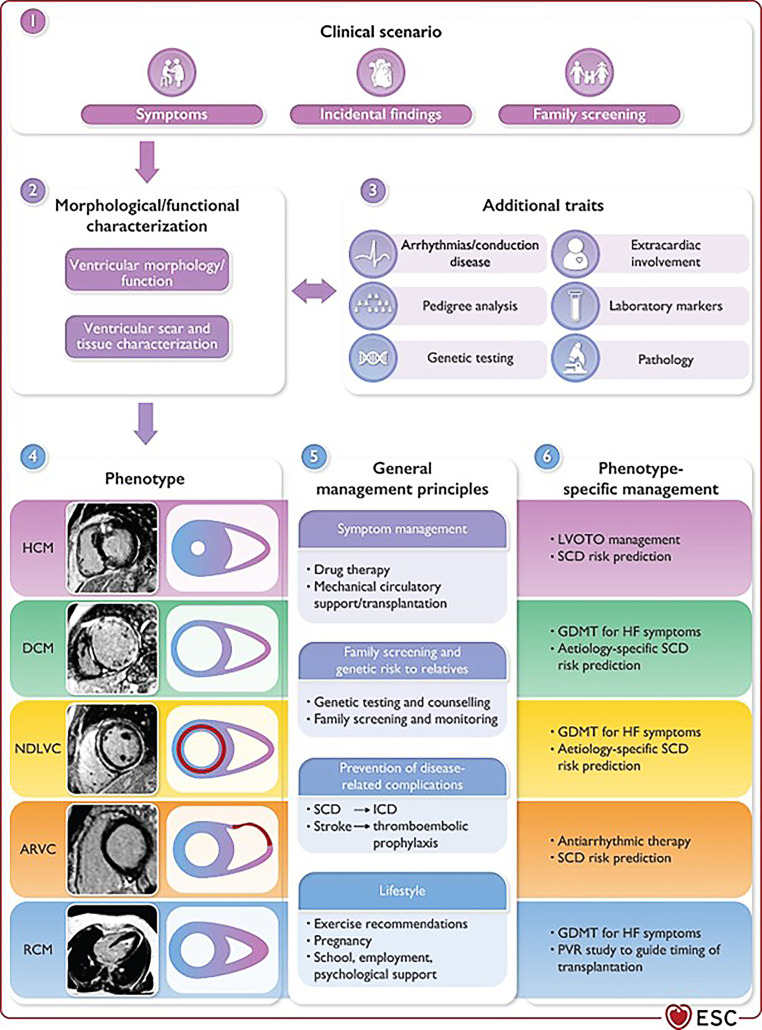
Update of the cardiomyopathy classification system to include uniform, describing diagnoses which lead to a conceptual framework for diagnosis and treatment [1] (*HCM* hypertrophic cardiomyopathy, *DCM* dilated cardiomyopathy, *NDLVC* non-dilated left ventricular cardiomyopathy, *ARVC* arrhythmogenic right ventricular cardiomyopathy, *RCM* restrictive cardiomyopathy, *SCD* sudden cardiac death, *ICD* implantable cardioverter-defibrillator, *LVOTO* left ventricular outflow tract obstruction, *GDMT* guideline-directed medical therapy, *HF* heart failure, *PVR* pulmonary vascular resistance). (Reproduced with the permission of Oxford University Press)

Innovative in this 2023 guideline is an update of the classification system to include new phenotypic descriptions and to simplify terminology, while simultaneously providing a conceptual framework for diagnosis and treatment (Fig. 1; [1]). The incentive for the classification update is that aetiology is vital to patient management and that a careful and consistent description of the morphological and functional phenotype is a crucial first step in the diagnostic pathway, while the final diagnosis will ideally describe aetiology alongside the phenotype, implying treatment options.

As the definition of dilated cardiomyopathy (DCM) had significant limitations for intermediate phenotypes with myocardial disease, a new entity has been introduced in the classification system. The non-dilated left ventricular cardiomyopathy (NDLVC) phenotype is defined as (a) the presence of non-ischaemic LV scarring or fatty replacement regardless of the presence of global or regional wall motion abnormalities, or (b) isolated global or regional LV hypokinesia without scarring [1]. This phenotype will include individuals that until now may have been described as having DCM (but without LV dilatation), arrhythmogenic left ventricular cardiomyopathy, left dominant arrhythmogenic right ventricular cardiomyopathy (ARVC) or arrhythmogenic DCM (but often without fulfilling the diagnostic criteria for ARVC). Identification of an NDLVC phenotype should trigger a multiparametric approach, combining clinical/imaging phenotype and genotype, that leads to a specific aetiological diagnosis with implications for treatment.

Not recommended according to the 2023 guidelines is the use of the umbrella term arrhythmogenic cardiomyopathy, as it lacks a morphological or functional definition consistent with the existing classification scheme [1]. A cardiomyopathy classification is also not recommended in Takotsubo syndrome, given the usually transient nature of abnormalities.

LV non-compaction (LVNC) is no longer considered to be a distinct cardiomyopathy in the general sense. Instead, it is seen as a phenotypic descriptive trait that can occur either in isolation or in association with other developmental abnormalities, ventricular hypertrophy, dilatation and/or systolic dysfunction. The term hypertrabecularisation is recommended instead of LVNC, particularly when the phenomenon is transient or clearly of adult onset.

The Netherlands Society of Cardiology (*Nederlandse Vereniging Voor Cardiologie*, NVVC) underscores the importance of using the updated classification system in the Netherlands.

### Integrated patient management

Diagnosis, evaluation and management of cardiomyopathy patients requires a systematic, individualised approach that delivers optimised care by a multidisciplinary and expert team. Central to the approach is not only the patient, but the whole family.

**Fig. 2:**
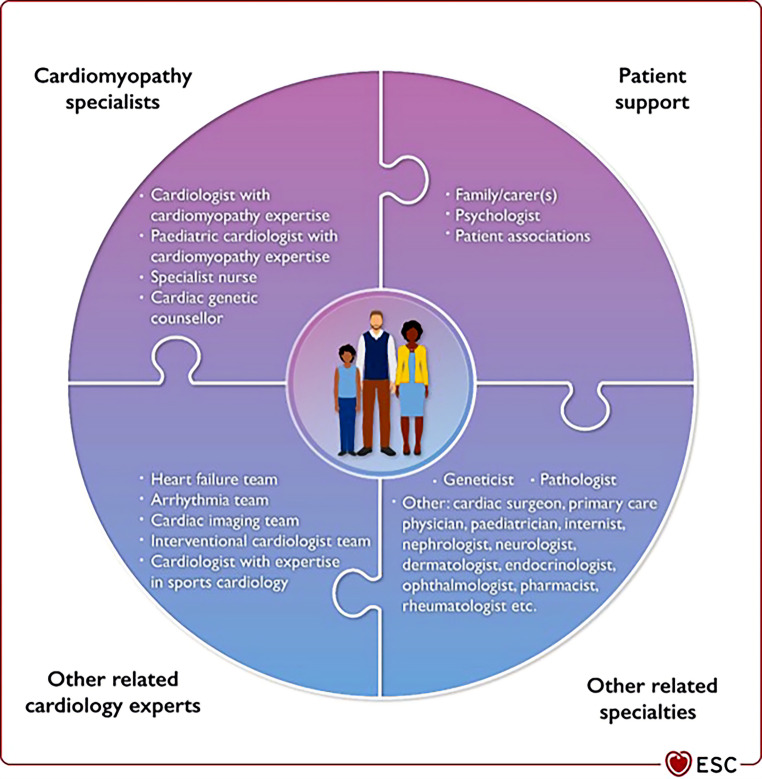
Integration of cardiogenetics into clinical cardiology practice requires expertise from different specialties, resulting in a multidisciplinary expertise team [1]. (Reproduced with the permission of Oxford University Press)

The integration of cardiogenetics into clinical cardiology practice requires expertise from different specialties: (a) adult and paediatric cardiologists with cardiogenetic expertise; (b) cardiac imaging specialists (technicians, cardiologists, radiologists); (c) clinical geneticists, specialist nurses and/or genetic counsellors with skills in family history taking, drawing pedigrees and patient/family management; (d) clinical psychologists to support patients and their relatives; (e) molecular geneticists and bioinformaticians to interpret the results of genetic investigations; (f) expert pathologists to interpret findings by endomyocardial biopsy (EMB) and autopsy of individuals dying from a suspected inherited and/or acquired cardiac condition. Moreover, patients’ associations should be actively involved in co-designing and conducting research as well as creating awareness for rare and very rare cardiac conditions among the population (Fig. 2; [1]).

In addition to genetic cardiomyopathies, patients with autoimmune diseases, systemic diseases and those receiving cancer treatment are all at higher risk of developing a cardiomyopathy, which also necessitates multidisciplinary consultation, including genetic counselling as part of the second hit paradigm.

**Fig. 3:**
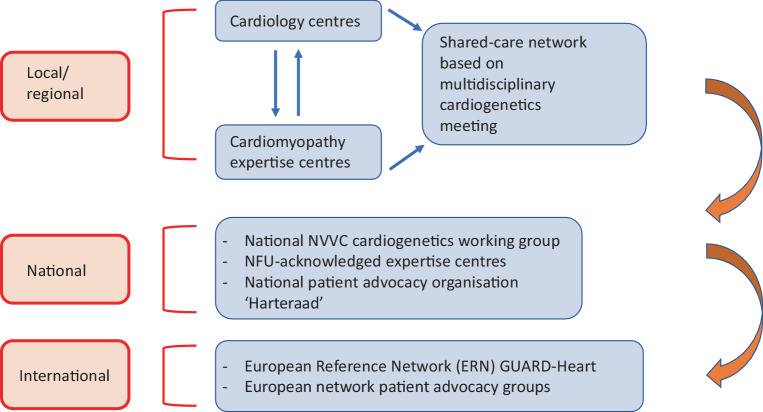
Schematic representation of local/regional, national and international shared-care expertise networks for Dutch cardiogenetics practice (*NVVC* Netherlands Society of Cardiology (*Nederlandse Vereniging voor Cardiologie*), *NFU* Netherlands Federation of University Medical Centres (*Nederlandse Federatie Universitair Medische Centra*))

A shared-care approach involving cardiomyopathy experts and general adult and paediatric cardiologists is strongly recommended. While cardiomyopathy expertise centres are essential for complex cases or care, general adult and paediatric cardiologists are key in diagnosis, follow-up and management of cardiomyopathy patients. The creation of local/regional/national/international networks supports knowledge dissemination and establishment of the shared-care approach. Figure 3 provides an overview of our national conduct. Protocolised transition of care from paediatric to adult cardiological services is additionally recommended.

### The patient pathway—aetiological work-up and follow-up

The cardiomyopathy management guidelines aim to create awareness of the possibility that a cardiomyopathy can underlie common cardiac symptoms and should be part of the differential diagnosis in clinical evaluation [1]. In most genetic cardiomyopathy forms, diagnostic evaluation is complicated by variable expression (different/overlapping cardiomyopathy phenotypes) and age-related and incomplete penetrance (varying age at onset and disease severity) within families. Identification of diagnostic clues and/or red flags is an important aspect in the work-up. The presence of acquired factors such as atrial fibrillation, previous chemotherapy, alcohol abuse or autoimmune disease does not exclude a genetic predisposition and does not exclude the requirement for genetic testing.

With every suspected or established cardiomyopathy, a systematic multiparametric evaluation is recommended, including clinical evaluation, pedigree analysis, 12-lead electrocardiogram (ECG), Holter monitoring, indicated laboratory tests, multimodality imaging and exercise testing upon indication. The separate parts of the multiparametric evaluation must be seen as pieces of the puzzle and interpreted in conjunction with the other findings.

Certain extracardiac symptoms and physical examination findings can raise suspicion of a specific aetiology (Table 6 in the guideline). ECG abnormalities are common and can precede an overt phenotype, thereby acting as important diagnostic clues and tools in risk assessment. Holter monitoring may reveal atrial fibrillation or ventricular arrhythmias. Specific arrhythmias can also aid in diagnosis and risk stratification.

Laboratory testing can reveal extracardiac conditions that cause or exacerbate ventricular dysfunction, secondary organ damage and may be of value for diagnostic, prognostic and therapeutic monitoring. Table 8 in the guideline provides a list of routine and additional laboratory tests for each phenotype. The added value of routine assessment in all patients is limited in our opinion. Therefore, in the Netherlands, the recommendation to perform these tests has been modified from ‘is recommended’ to ‘should be considered’.

Non-invasive imaging modalities are the backbone of diagnosis and follow-up in cardiomyopathy patients. Modality choice and timing of assessment should be guided by a trade-off in yield of actionable results, technique advantages and limitations, costs and safety (Fig. 6 and Table 9 in the guideline). These decisions are preferably based on consultation in the multidisciplinary team meeting. Cardiac magnetic resonance imaging (CMR) has a prominent role in the evaluation of cardiomyopathy, especially in the first aetiological work-up (class I recommendation) [1].

EMB in experienced centres with expert pathological evaluation should be considered in diagnosis and management when there is a clinical suspicion of myocardial inflammation, infiltration, or storage disease that cannot be identified by other means. Further studies on the role of genomics and transcriptomics in cardiac samples to identify novel therapeutic targets in genetic and/or acquired cardiomyopathies are needed [2]. EMB will also be required in upcoming gene therapy studies to confirm target engagement.

Lifelong follow-up is indicated in cardiomyopathy patients. Routine follow-up is recommended every 1–2 years. Follow-up frequency and investigations should be guided by symptoms and phenotypic stability trend. Hence, individual decision making is based on multidisciplinary team consultation. The ESC guidelines recommend performing an ECG, Holter monitoring and an echocardiogram at routine follow-up. Exercise testing (ergometry or cardiopulmonary exercise testing) can be performed less frequently. Although we agree with the guideline that serial CMR follow-up every 2–5 years (depending on initial severity and clinical course) can assist in evaluating disease progression and/or therapy effect, the availability, use of resources and costs should also be considered. Therefore, in the Netherlands, we recommend that the frequency of serial CMR follow-up be based on actionable yield instead of being performed routinely in all patients. Also, echocardiography and CMR do not have to be performed at the same time during the follow-up period.

In summary, for clinical practice in the Netherlands the first clinical work-up should be as extensive as needed to obtain an aetiological diagnosis, as the specific aetiology guides the appropriate treatment options for each specific cardiomyopathy. Follow-up should be directed by the spectrum of initial phenotype severity, symptoms and clinical course. Decisions should be based on (expert) multidisciplinary team consultation and executed in shared-care networks, preferably supported by sharing of clinical and imaging data to enable multidisciplinary and cross-institution care.

### The patient pathway—genetic testing and family screening

Evaluation of a three- to four-generation family pedigree and family history (sudden cardiac death, heart failure, pacemaker/defibrillator, signs of systemic disease such as skeletal muscle disease, etc.) is strongly recommended to aid in diagnosis, provide clues on the inheritance pattern (Table 5 in the guideline) and identification of family members at risk [1].

The variable expression and age-related, incomplete penetrance in cardiomyopathies could be explained by genetic variant heterogeneity, external factors such as hypertension in hypertrophic cardiomyopathy (HCM) or exercise in ARVC and/or the contribution of additional common genetic variants. Evidence of modulation by common genetic variants in addition to rare pathogenic variants, or even on their own, has recently been provided (Fig. 8 and references 182/183 in the guideline).

Genetic testing is recommended in cardiomyopathy index patients, anticipating benefits regarding (a) diagnosis, (b) prognosis, (c) treatment options and (d) reproductive management. Even if there is no direct patient benefit, if relatives can possibly benefit (by cascade genetic testing and thereby identification of relatives at risk), genetic testing in the index patient is recommended.

In families where a likely pathogenic (LP) or pathogenic (P) variant (variant class 4 or 5, see Tab. 1 for variant classification and Fig. 11 in the guideline for more information) is found, family members harbouring the variant should subsequently undergo repeated cardiac evaluation. Which tests are performed during evaluation depends on the underlying genotype. Family members without the variant can be discharged. In families where no genetic substrate is found, first-degree family members (parents/siblings/children) should be referred for cardiac evaluation. Genetic cascade screening is not recommended when genetic testing in the index patient only reveals a variant or variants of unknown significance (VUS, variant class 3). Segregation analysis (genotype-phenotype correlation analysis in families) of a VUS can help in the interpretation of its significance. The polygenic risk score, now only performed in a research setting, is a genetic test that evaluates many contributing variants across the genome and calculates an aggregated risk and can thus be of future importance in diagnostic testing.

**Table 1:** Genetic variant classification

Variant classification	Variant effect	Genetic test result
Variant class 1	Benign	Not reported
Variant class 2	Likely benign	Not reported
Variant class 3	Unknown significance	Reported to physician
Variant class 4	Likely pathogenic	Reported to physician
Variant class 5	Pathogenic	Reported to physician

Genetic testing should always be accompanied by genetic counselling by an appropriately trained healthcare professional. Chapter 6.8 and Table 13 in the guideline provide more information on (recommendations for) genetic testing, counselling and pre-natal genetic testing.

This approach translates into a cardiological screening indication for family members with: (a) the LP or P variant (class 4 and 5 variants respectively) found in the index patient, (b) no genetic screening in the index patient or when no LP/P genetic variant was identified, or (c) segregation purposes in case of a VUS (class 3 variant).

Cardiological evaluation should include electrocardiography and cardiac imaging. Holter monitoring should be included in most cardiomyopathy forms when there are phenotypic abnormalities. Further analysis is performed if indicated. The follow-up frequency depends on multiple individual factors (age, cardiomyopathy form, family history, lifestyle, precipitated individual risk etc.). The proposed general follow-up frequency is every 1–5 years.

Screening of children who are possibly at risk should be contextualised by family history, specific genetic variant or cardiomyopathy form and based on multidisciplinary cardiogenetic meetings that include paediatric cardiologists (expert opinion). The mainstay according to the guidelines is that screening, whether genetic or cardiological, should be in the child’s best interests and have an impact on management, lifestyle and/or ongoing clinical testing.

### Management of cardiomyopathy patients

Generic clinical management recommendations for heart failure treatment are provided in the 2021 ESC guidelines and the 2023 focused update [3, 4]. General applicable cardiac resynchronisation therapy (CRT) indications are formulated in the 2021 ESC guidelines on cardiac pacing and the NVVC endorsement statement [5, 6]. Particularly in genetic DCM, weaning of guideline-directed medical therapy (GDMT) after improvement or recovery of LV function may cause deterioration again [7]. In addition to the guideline recommendations for cardiac transplantation and left ventricular assist device (LVAD) therapy, for clinical practice in the Netherlands we include the recommendations from the Dutch Society of Thoracic Surgery (*Nederlandse Vereniging voor Thoraxchirurgie*, NVT)/NVVC consensus document on LVAD therapy (*Consensus Document LVAD therapie Werkgroep Mechanical Circulatory Support (MCS)*, www.nvvc.nl/richtlijnen).

Preventive medical therapy in asymptomatic LV dysfunction, early disease forms, or even in genotype-positive/phenotype-negative individuals is challenging, as evidence evaluating the effect on disease development/progression is lacking [8]. First-line GDMT may be considered in patients with early LV cardiomyopathy to prevent progression (e.g. angiotensin-converting enzyme inhibitors, angiotensin receptor blockers, beta blockers and mineralocorticoid receptor antagonists, class IIb Level of Evidence (LOE) C) [1]. Medical treatment of (early) RV dysfunction is even more difficult considering the evidence gap. We propose the same measures (first-line GDMT may be considered) as in early LV cardiomyopathies, with patient-tailored decisions on the medication sequence.

### Management of arrhythmias

Considering anticoagulation with any type of atrial fibrillation or atrial flutter/tachycardia in cardiomyopathies is key. The CHA_2_DS_2_-VASc score has not been validated for cardiomyopathy patients. HCM, cardiac amyloidosis and restrictive cardiomyopathy forms have a specifically increased cardioembolic risk [9, 10]. Therefore, in the case of atrial arrhythmias in HCM, cardiac amyloidosis (both class I recommendation, LOE B) and restrictive cardiomyopathy forms (class IIa recommendation, LOE B), it is recommended that prophylactic anticoagulation be initiated [1]. For DCM, NDLVC and ARVC the anticoagulation recommendation is based on the CHA_2_DS_2_-VASc score. There are no randomised controlled trials on anticoagulation type in cardiomyopathy; recommendations are the same as those for the general population.

A rhythm control strategy, including early catheter ablation, in cardiomyopathies with atrial arrhythmias is usually preferred over rate control, as it reduces heart-failure-related morbidity (see also Table 15 in the guideline for recommendations for each specific cardiomyopathy phenotype). If rhythm control fails, a rate control strategy is preferred. Integrated, structured approaches are outlined in the 2024 ESC guidelines on atrial fibrillation and the 2021 ESC guidelines on heart failure [3, 11].

Ventricular arrhythmias increase morbidity and mortality in cardiomyopathy patients. The acute and long-term treatment of ventricular arrhythmias for each specific cardiomyopathy phenotype is outlined in Chapter 7 of the guideline and generic treatment is outlined in the 2022 ESC guidelines on the management of ventricular arrhythmias [12].

Risk calculators, indicating the individual risk of malignant ventricular arrhythmias and sudden cardiac death, thereby aiding in clinical decision making on implantable cardioverter-defibrillator (ICD) implantation for primary prevention, exist for HCM and ARVC and gene-specific risk calculators for phospholamban (*PLN*) and lamin A/C (*LMNA*) [13, 14]. A limitation of the risk calculators, with the exception of the HCM calculator, is that no cut-off values are defined and that not sudden cardiac death but, amongst others, appropriate ICD therapy was used as an endpoint. High-risk features for each cardiomyopathy phenotype (HCM, DCM, ARVC) and gene-specific (Filamin C (*FLNC*), desmoplakin (*DSP*), RNA binding motif protein 20 (*RBM20*)) high-risk features are elaborated on in Chapter 7 of the ESC guideline. In addition to the ICD recommendations in the ESC guidelines, including those for specific cardiomyopathy phenotypes, in clinical practice in the Netherlands we include the recommendations from the Dutch guideline on primary prevention in non-ischaemic cardiomyopathy (NICM) (*Indicatierichtlijn primaire preventie ICD plaatsing bij NICM 2023*, www.nvvc.nl/richtlijnen). According to this national guideline, ICDs for primary prevention are only indicated in NICM when (a) the LV ejection fraction is ≤ 35%, (b) the New York Heart Association class is II-III after GDMT, (c) the patient is not suitable for CRT and (d) there is late gadolinium enhancement on CMR. Exceptions to these limitations are made for those patients carrying genetic variants in the high-risk arrhythmic genes *PLN, FLNC, RBM20, DSP* and/or *LMNA*.

### Specific cardiomyopathy management recommendations

The 2023 ESC guidelines on the management of cardiomyopathies are new and provide background knowledge, an overview and recommendations on the general topic of cardiomyopathies. Previously, only the 2014 ESC guidelines for HCM existed. Chapter 7 of the ESC guideline provides insight into and specific recommendations for the different cardiomyopathy subtypes, with an update of the guideline information for HCM and new guidelines for the other categories.

With regard to the management of HCM with left ventricular outflow tract (LVOT) obstruction, there has been a significant change in treatment options in the Netherlands since 2024. By convention, obstructive HCM is defined by the presence of an LVOT gradient ≥ 30 mm Hg, where an LVOT gradient ≥ 50 mm Hg is haemodynamically significant and is the threshold for specific therapy. Patients with obstructive HCM who remain symptomatic and have an LVOT gradient ≥ 50 mm Hg and an LV ejection fraction ≥ 55% despite treatment with non-vasodilatation beta blockers or non-dihydropyridine calcium channel blockers or who do not tolerate these drugs are possible candidates for treatment with selective myosin inhibitors. Mavacamten (a selective cardiac myosin inhibitor) was approved for obstructive HCM by the European Medicines Agency in 2023 and by the Dutch National Healthcare Institute (*Zorginstituut Nederland*, ZiN) in 2024 [15]. In patients treated with mavacamten, CYP2C19 should be genotyped. Treatment with mavacamten must be monitored according to the Summary of Product Characteristics, which requires frequent echocardiographic evaluation. Evaluation of possible candidates, initiation of treatment with mavacamten and monitoring are currently confined to expertise centres with Netherlands Federation of University Medical Centres (*Nederlandse Federatie van Universitair Medische Centra*, NFU) accreditation for HCM and centres that have participated in selective myosin-inhibitor trials, as required by ZiN. Tables 18 and 19 in the guideline provide recommendations for general measures and drug therapy in patients with obstructive HCM. Table 20 in the guideline provides recommendations for invasive therapy in patients with obstructive HCM. Because the LVOT and the mitral valve have specific anatomical features, some patients with HCM will be more suitable candidates for septal myectomy than alcohol septum ablation. All patients should be assessed by experienced multidisciplinary teams before intervention, as morbidity and mortality are highly dependent on the available level of expertise. Invasive therapy should therefore only be performed by experienced operators who are part of a multidisciplinary team in cardiomyopathy expertise centres and after consultation in the cardiogenetics meeting. Symptomatic patients with non-obstructive HCM are managed in accordance with the current heart failure guidelines [3]. Chest pain can be treated with either beta blockers or non-dihydropyridine calcium channel blockers. Ranolazine, recommended in Table 22 of the guideline, is not available in the Netherlands. Specific Dutch guidelines on genetic screening in HCM are provided at: https://richtlijnendatabase.nl/richtlijn/hypertrofische_cardiomyopathie_hcm.

In patients with NDLVC, we are of the opinion that it is usually not necessary to perform Holter monitoring annually. The frequency can be adjusted at the discretion of the treating physician. Holter monitoring may therefore be considered every 1–5 years, pending the underlying findings.

The recommendation to perform Holter monitoring is not applicable to ARVC patients with an ICD. Furthermore, in the Netherlands, we have extensive experience with sotalol as an anti-arrhythmic treatment option for ARVC patients and, therefore, we include a recommendation for sotalol in addition to other recommendations in Table 28 of the guideline. Sotalol is a very potent drug in the prevention of ventricular tachycardias in ARVC patients and should be considered if beta-blocker therapy fails (Tab. 2).

**Table 2:** European Society of Cardiology (*ESC*) guideline recommendations adopted into clinical practice in the Netherlands

ESC guideline	Class	LOE	Table	Update for Dutch clinical practice—recommendation
*Recommendation Chapter 6.6*				
Routine (first-level) laboratory tests *are recommended* in all patients with suspected or confirmed cardiomyopathy to evaluate aetiology, assess disease severity and aid in detection of extra-cardiac manifestations and assessment of secondary organ failure	I	C	8	Routine (first-level) laboratory tests *should be considered* in all patients with suspected or confirmed cardiomyopathy to evaluate aetiology, assess disease severity and aid in detection of extra-cardiac manifestations and assessment of secondary organ failure
*Recommendation Chapter 6.7.3*				
Contrast-enhanced CMR should be considered *in all patients *with cardiomyopathy during follow-up (text: every 2–5 years) to monitor disease progression and aid in risk stratification and management	IIa	C	5	Contrast-enhanced CMR should be considered *based on actionable yield *during follow-up (text: every 2–5 years) to monitor disease progression and aid in risk stratification and management
*Recommendation Chapters 6.10.2.2 and 6.10.2.3*				
ESC guideline recommendations for cardiac transplantation and LVAD therapy	–	–	9/10	Inclusion of recommendations from the NVT/NVVC consensus document on LVAD therapy
*Recommendation Chapters 6.10.5 and 7*				
ESC guideline recommendations for ICD implantation in cardiomyopathy patients, including those for each specific cardiomyopathy phenotype	–	–	–	Inclusion of recommendations from the Dutch guideline on primary prevention in non-ischaemic cardiomyopathy
*Recommendation Chapter 7.1.4.2*				
Ranolazine may be considered to improve symptoms in patients with angina-like chest pain even in the absence of LVOT obstruction or obstructive coronary artery disease	IIb	C	22	Ranolazine is not available in the Netherlands
*Recommendation Chapter 7.3.1.4*				
Annual Holter monitoring is recommended in patients with NDLVC or when there is a change in clinical status, to aid in management and risk stratification	I	C	25	The frequency can be adjusted at the discretion of the treating physician. However, Holter monitoring should be performed every 1–5 years
*Recommendation Chapter 7.4.1.4*				
Annual Holter monitoring is recommended in ARVC patients to aid in diagnosis, management and risk stratification	I	C	27	The recommendation to perform Holter monitoring is not applicable for ARVC patients with an ICD
*Recommendation Chapter 7.4.4.1*				
No recommendation on sotalol	–	–	28	Sotalol should be considered as anti-arrhythmic therapy in ARVC patients after regular beta-blocker therapy has failed
*Recommendation Chapter 8.3*				
In patients aged < 65 years with a first-degree relative with a cardiomyopathy, it is recommended that an ECG and TTE be performed before non-cardiac surgery, regardless of symptoms	I	C	33	An ECG is usually routinely performed in work-up of non-cardiac surgery; a TTE should be considered if the ECG is abnormal and/or the patient displays symptoms

*LOE* level of evidence, *CMR* cardiac magnetic resonance, *LVAD* left ventricular assist device, *NVT* Dutch Society of Thoracic Surgery (*Nederlandse Vereniging voor Thoraxchirurgie*), *NVVC* Netherlands Society of Cardiology (*Nederlandse Vereniging voor Cardiologie*), *ICD* internal cardioverter-defibrillator, *LVOT* left ventricular outflow tract, *NDLVC* non-dilated left ventricular cardiomyopathy, *ARVC* arrhythmogenic right ventricular cardiomyopathy, *ECG* electrocardiogram, *TTE* transthoracic echocardiogram

## Discussion

The 2023 ESC guidelines on the management of cardiomyopathies are the first of their kind, encompassing the diagnostic work-up, genetic screening, risk stratification and management of cardiomyopathy patients and their families in general.

A new phenotypic classification system for cardiomyopathies has been developed. This new taxonomy proposes a more personalised approach to monitoring follow-up and guiding specific treatment tailored to the underlying aetiology. A deep phenotyping approach is proposed with an important role for CMR and genetic testing in the aetiological work-up, risk stratification and follow-up of cardiomyopathy patients.

Cardiogenetics is becoming increasingly important as clinical implications expand for the patient and (future) relatives with gene-specific care pathways and treatment options. Cardiogenetics is a multidisciplinary field that operates in shared-care networks with referral hospitals (Figs. 2 and 3). Optimal organisation of shared-care networks with cardiomyopathy expertise centres on a local, regional, national and international level ensures optimal quality of care for cardiomyopathy patients. This guideline also aids in providing a framework for uniform steps in the multiparametric diagnostic approach and follow-up of cardiomyopathy patients.

In addition to a brief summary of relevant guideline information, we have presented an overview of ESC guideline recommendations not suitable for cardiology practice in the Netherlands and have provided alternative recommendations. This also implies that all other recommendations made in the 2023 ESC guidelines on the management of cardiomyopathies are endorsed by the NVVC.

## Conclusion

This contextualisation of the 2023 ESC guidelines on the management of cardiomyopathies serves as an overview with relevant recommendations for daily clinical practice in the Netherlands, while also pointing out sources of more in-depth knowledge on (the management of) cardiomyopathies.
